# Systemic anticancer therapy at the end of life: real-world insights from a tertiary oncology center in Israel

**DOI:** 10.1093/oncolo/oyaf066

**Published:** 2025-05-16

**Authors:** Renana Barak, Esraa Safadi, Alla Nikolaevski-Berlin, Noa Soback, Ido Wolf, Barliz Waissengrin

**Affiliations:** Oncology Institute, Tel Aviv Sourasky Medical Center, Tel Aviv, Israel; Faculty of Medicine, Tel Aviv University, Tel Aviv, Israel; Oncology Institute, Tel Aviv Sourasky Medical Center, Tel Aviv, Israel; Faculty of Medicine, Tel Aviv University, Tel Aviv, Israel; Oncology Institute, Tel Aviv Sourasky Medical Center, Tel Aviv, Israel; Faculty of Medicine, Tel Aviv University, Tel Aviv, Israel; Oncology Institute, Tel Aviv Sourasky Medical Center, Tel Aviv, Israel; Faculty of Medicine, Tel Aviv University, Tel Aviv, Israel; Oncology Institute, Tel Aviv Sourasky Medical Center, Tel Aviv, Israel; Faculty of Medicine, Tel Aviv University, Tel Aviv, Israel

**Keywords:** systemic anticancer therapy, palliative care, good death, end of life, aggressive care

## Abstract

**Background:**

Aggressive end-of-life (EOL) care, such as systemic anticancer therapy (SACT) for advanced cancer patients, represents a potential indicator of low-quality care that may deviate from the primary palliative objective of treatment.

**Methods:**

A retrospective study analyzed consecutive patients with advanced cancers treated at a tertiary oncology center in Israel from January 2019 to December 2022. Demographic and clinical data were examined, with a focus on intravenous (IV) oncologic treatment administration rates at 30 and 90 days before death.

**Results:**

The study included 1851 patients who received IV oncologic medications and died during 2019-2022. The median age at death was 69 years, with 51.3% (951) being men. Systemic anticancer therapy administration rates were 36% (666 patients) in the last 30 days and 67.6% (1252 patients) in the last 90 days prior to death. Chemotherapy was the most common EOL medication (58%). Higher EOL SACT rates were associated with younger age, better ECOG performance status, shorter disease duration, and specific tumor origins, particularly breast cancer. Conversely, gender, marital status, and ethnicity showed no significant correlation with EOL treatment use.

**Discussion:**

Our data provide insight into current practice adopted by healthcare professionals regarding EOL treatment administration in Israel. A positive EOL experience is a significant goal in the oncology clinic, yet our findings demonstrate high rates of aggressive EOL care and may highlight the necessity for regulatory and educational changes within the healthcare system.

**Implications for Practice**

This study reveals high rates of systemic anticancer therapy administration near the end of life in an Israeli tertiary cancer center, particularly for younger patients, with short duration of disease and good performance status. The findings underscore the critical need for further investigation into underline causes of this practice. We suggest that future research should focus on structured guidelines for treatment discontinuation, better predictive models for identifying patients who might benefit from continued therapy and enhanced integration of palliative care services. Altogether, medical oncologist should strive to align treatment decisions with individual patients’ preferences and goals, thereby enhancing both quality of life and death.

## Introduction

In recent decades, substantial advancements have reshaped cancer treatment, particularly in the metastatic setting. Novel systemic anticancer therapy (SACT), including chemotherapy, antibody-drug conjugates, biologic targeted therapies, and immune checkpoint inhibitors (ICIs), has shown improved patient survival and quality of life.^1,2^ However, the primary therapeutic focus remains palliative, emphasizing symptom control and overall well-being.^3,4^

Near the end of life (EOL), the use of molecular targeted therapies and immunotherapy is increasing, largely due to their favorable side effect profiles, ease of administration, and potential for long-term benefits compared with traditional chemotherapy.^5-8^ However, these therapies exhibit a distinct but significant toxicity profile, with increased rates of delirium and opioid use observed among patients treated with molecular therapies and ICIs, respectively, at the EOL.^9^ Other studies suggested that the optimism surrounding these treatments can lead patients and physicians to overestimate their efficacy.^7,10,11^

End-of-life oncologic treatments have been associated with adverse outcomes, including increased use of urgent medical services, delayed EOL discussions, reduced hospice enrollment, financial toxicity, and negative impacts on quality of life.^5,7,12^ While these concerns have been well-documented for chemotherapy, recent evidence highlights similar risks for ICI and targeted therapies.

In response, the American Society of Clinical Oncology (ASCO) introduced the “Choosing Wisely” campaign in 2013, providing guidelines to discourage chemotherapy use in late-stage illness. Subsequent updates in 2014 and 2021 expanded these recommendations to include immunological and targeted therapies.^13-15^ Despite these efforts, a significant proportion of advanced cancer patients continue to receive oncologic treatment near the EOL, with up to 38% receiving it within 1 month of death.^4,6,12,16-22^

Robust evidence supports the benefits of early palliative care, particularly in managing symptoms and improving quality of life for cancer patients. Prioritizing palliative care alongside treatment decision-making can help steer EOL care toward a less aggressive trajectory. Some studies even suggest a modest survival benefit with best supportive care alone at EOL.^23,24^ Yet, the appropriateness of early treatment withdrawal is not universally established, as some patients might still derive therapeutic advantages from less intensive medical approaches.

In Israel, the public health system subsidizes medical and oncologic treatments, including biologic therapies and ICI, which are part of the national healthcare basket for advanced cancers.^25-27^ However, the difficulty of predicting patient survival^28-30^ and the challenge of discontinuing active treatment may encourage continued use of these therapies at the EOL. Additionally, while home hospice services have been introduced as part of a national program to improve EOL care, their utilization remains limited. According to 2016 Ministry of Health data, only 6.7 per 1000 individuals aged 65 and over received care in home hospice units, with cancer accounting for 65% of diagnoses.^25^ This highlights a significant gap between service availability and patient needs.

This study aims to explore trends in SACT use at the EOL in Israel, considering recent advancements in treatment options and the evolving healthcare landscape. By analyzing these patterns, we seek to better understand the factors influencing treatment decisions and identify opportunities to optimize EOL care within the healthcare system.

## Methods

### Study population

We conducted an observational, single center, retrospective cohort study. The study included patients aged 18 years and older with advanced solid malignancies who were treated at the Tel Aviv Sourasky Medical Center (TASMC) oncology department or day care unit and died between 2019 and 2022. Patients were eligible for inclusion if they received intravenous oncologic treatments such as IV chemotherapy, ICIs, or monoclonal antibodies. Patients enrolled in phase 1-3 clinical trials were also included. The exclusion criteria involved administration of oral or intramuscular oncologic treatments at the EOL, as with these routs of administration we were unable to accurately monitor the dates of treatment administration or cessation at home or other ambulatory care settings. Consequently, excluded treatments primarily comprised hormonal therapies and small molecule inhibitors.

### Data

Demographic and clinical data were drawn from electronic medical records. Information on age, gender, ethnicity, marital status, oncological diagnosis, ECOG performance status recorded during the most recent oncology clinic visit, duration of disease (from date of diagnosis, based on biopsy date until death), date of death (based on the Population and Immigration Authority), and date and type of last treatment before death (ICI, chemotherapy protocol, biological treatments, clinical trial) were collected. The study was approved by the Helsinki Committee for Clinical Research at TASMC (Helsinki number 1031-20).

### Statistical analysis

All explanatory variables (demographic and clinical characteristics of the patients) were characterized by appropriate descriptive measures. We examined the relationship between each of the explanatory variables and receiving oncology treatment in the last 30 days of life and in the last 90 days of life by chi-square test for independence. Logistic regression models were performed to investigate the influence of patients’ demographic (age, gender, marital status, nationality) and clinical characteristics (diagnosis, disease duration, treatment type, ECOG performance status) on the probability of receiving IV SACT in the last 30 and 90 days of life. Continuous variables, such as age at diagnosis and disease duration, were converted into categorical inputs for the regression model, with cutoffs determined according to quartiles of the study population (ie, <25%, 25%-74%, and ≥75%). Among patients receiving chemotherapy at EOL, logistic regression model was performed to investigate the impact of same patients’ clinical and demographic characteristics on the probability of receiving monotherapy regimen during the last 30 days of life. Statistical analysis was performed by SAS 9.4 software.

## Results

Out of 21 040 patients that were treated at the TASMC oncology department or day care unit between 2019 and 2022, 1851 patients met the inclusion criteria. The baseline characteristics of these eligible patients are detailed in Table 1. Among them, 951 (51.3%) were men, the median age at death was 69 years (range 21-102), and the majority of patients were Jewish 1805 (97.5%). At the most recent oncology clinic visit, 1497 patients (80.8%) had an ECOG score of 0-2. The most prevalent oncological diagnoses were gastrointestinal (GI) malignancies with 708 cases (38.2%), followed by lung cancer with 327 cases (17.7%), central nervous system (CNS) tumors 141 (7.6%), and breast cancer 137 (7.4%). The median duration of disease, measured from the date of diagnosis (biopsy date) to death, was 1.6 years. The upper quartile included patients with a disease duration exceeding 3.4 years, while the lower quartile comprised patients with a disease duration shorter than 9.6 months. This distribution was subsequently used for subgroup comparisons in the multivariate regression models. Chemotherapy was the most common last treatment administered, occurring in 1057 cases (57.1%), followed by ICI in 278 cases (15%) and monoclonal antibodies (mAB) 188 (10.2%). Notably, only 87 (4.7%) were enrolled in a clinical trial, of whom nearly two-thirds (59 patients) participated in a phase 1 trial, and 1252 patients (67.6%) and 666 patients (36%) received systemic anticancer treatment in the last 90 and 30 days before death, respectively.

**Table 1.** Baseline and clinical characteristics of the study population (*N* = 1851).

Variable	Value	Count (%)
Age at diagnosis (median [range], years)		67 (18-101)
Age at death (median [range], years)		69 (21-102)
Gender	Male	951 (51.3)
Marital status	Single	152 (8.2)
	Married	1168 (63.1)
	Divorced	308 (16.64)
	Widower	223 (12)
Nationality	Jewish	1805 (97.5)
	Arab	46 (2.5)
ECOG performance status^a^	0-2	1496 (80.8)
	3-4	308 (16.6)
Oncologic diagnosis	GI malignancies	708 (38.25)
	Lung malignancies	327 (17.67)
	CNS malignancies	141 (7.62)
	Breast malignancies	137 (7.4)
	GU malignancies	132 (7.13)
	Gynecologic malignancies	124 (6.7)
	Head and neck malignancies	58 (3.13)
	Other	224 (12.1)
Type of last treatment	Chemotherapy	1057 (57.1)
	Immune-Checkpoint Inhibitors	278 (15)
	Monoclonal Antibodies	188 (10.2)
	Chemotherapy + Monoclonal Antibodies	158 (8.5)
	Clinical Trial	87 (4.7)
	Chemotherapy + Immune-Checkpoint Inhibitors	58(3.1)
	Immune-Checkpoint Inhibitors + Monoclonal Antibodies	25 (1.4)
Duration of disease (median [Q1, Q3], years)^b^		1.6 (0.8, 3.4)
Systemic anticancer treatment administration at EOL	30 days before death	666 (36%)
	90 days before death	1252 (67.6%)

An overview of the cohort’s demographic and clinical characteristics.

Other: skin, sarcoma, neuroendocrine tumors.

Abbreviations: EOL, end of life; GI, gastrointestinal; GU, genitourinary; CNS, central nervous system; Q1, first quartile, Q3, third quartile.

^a^ECOG performance status score recorded during the most recent oncology clinic visit.

^b^Duration of disease, from diagnosis until death.

### Characteristics of patients receiving treatment at EOL

Among 666 patients (36%) treated with systemic anticancer treatment in the last 30 days before death and 1252 patient (67.6%) treated with SACT during their last 90 days, similar distribution of baseline characteristics was seen, as detailed in Table 2. In 30-day time point, 50.7% (338) of treated patients were men, 586 (88%) had an ECOG score of 0-2, and 306 (46%) had disease duration of less than one year. In 90-day time point, 649 (51.8%) were men, 1034 (82.5%) had an ECOG score of 0-2, and disease duration was less than a year for 510 (41%) patients. Looking at disease duration, almost 90% (510) of patients with disease duration of less than a year received SACT in the last 90 days and 52.7% (306) were treated during the last month before death, while patients with disease duration longer the 3.5 years had substantially lower rates of SACT administration at EOL (50% and 6% at 90- and 30-day time points, respectively). Univariate analysis demonstrated significant associations between ECOG performance status score, disease duration, and tumor origin and treatment administration at both 30 and 90-day time points (*P* <.05 for all). In contrast, demographic variables such as gender, marital status, and ethnicity were not associated with the administration of anticancer treatment at the EOL.

**Table 2.** Systemic anticancer therapy administration at end of life, comparisons in 30 and 90 day time points.

Variable	Value	SACT use in the last 30 days, *N* (%)	*P*	SACT use in the last 90 days, *N* (%)	*P*
Full sample		666 (36)		1252 (67.6)	
Age at diagnosis	<55	141 (36.1)	.16	268 (68.7)	.74
	55-74	365 (38)		653 (68)	
	≥75	145 (32.7)		294 (66.4)	
Gender	Male	338 (35.5)	.69	649 (68.2)	.58
	Female	328 (36.4)		603 (67)	
Marital status	Single	59 (38.8)	.37	102 (67.1)	.07
	Married	420 (36)		801 (68.6)	
	Divorced	117 (38)		215 (69.8)	
	Widower	70 (31.4)		134 (60)	
Nationality	Jewish	649 (36)	.87	1222 (67.7)	.75
	Arab	17 (37)		30 (65.2)	
ECOG performance status^a^	0-2	586 (39.1)	**<.0001**	1034 (69.1)	**.046**
	3-4	80 (26)		195 (63)	
Oncologic diagnosis	GI malignancies	260 (36.7)	**<.004**	491 (69.3)	**.034**
	Lung malignancies	126 (38.5)		222 (67.9)	
	Breast malignancies	56 (40.9)		93 (67.9)	
	Gynecologic malignancies	45 (36.3)		88 (71)	
	CNS malignancies	29 (20.6)		95 (67.4)	
	GU malignancies	39 (29.5)		72 (54.5)	
	Head and neck malignancies	24 (41.4)		45 (77.6)	
	Other	87 (38.8)		146 (65.2)	
Type of last treatment	mAB	50 (26.6)	**.0135**	128 (68.1)	.62
	CT	386 (36.5)		716 (67.7)	
	ICIs	118 (42.4)		193 (69.4)	
	Clinical Trial	28 (32.2)		55 (63.2)	
	CT + mAB	56 (35.4)		99 (62.7)	
	ICIs + mAB	5 (20)		19 (76)	
	CT + ICIs	23 (39.7)		42 (72.4)	
Duration of disease^b^, years	<1	306 (52.7)	**<.0001**	510 (87.8)	**<.0001**
	1-3.4	251 (30.5)		520 (63.3)	
	≥3.5	109 (5.9)		222 (50)	

Univariate analysis of clinic-demographic variables and receipt treatment in 30 and 90 days before death.

Other: skin, sarcoma, neuroendocrine tumors.

Abbreviations: GI, gastrointestinal; GU, genitourinary; CNS, central nervous system; CT, chemotherapy; ICIs, immune checkpoint inhibitors; mAB, monoclonal antibodies. Values that are statistically significant (*P* ≤ 0.05) are indicated in bold.

^a^ECOG performance status score recorded during the most recent oncology clinic visit.

^b^Duration of disease, from diagnosis until death.

### Treatment modalities and protocols at EOL

Chemotherapy (CT) was the most commonly administered treatment, utilized by 386 patients (58%) and 716 patients (57.2%) in 30- and 90-day time points, respectively. At 30-day time point, chemotherapy was followed by ICI (17.7%, *n* = 118), CT combined with mAB (8.4%, *n* = 56), mAB alone (7.5%, *n* = 50), and participation in clinical trials (4.2%, *n* = 28). Similar distribution was observed at 90 the day time point, except for monoclonal antibodies regimens which were more frequently administered without chemotherapy (10.2%, *n* = 128) compared with chemotherapy (7.9%, *n* = 99). However, the type of treatment was significantly associated with treatment administration only at 30-day time point (*P* =.0135).

Seventy percent (*n* = 269) of chemotherapy-based regimens administered during the last month before death consisted of multiagent, while only 30% (*n* = 117) were single agent protocols. A multivariate logistic regression was conducted to assess the influence of clinical variables on the likelihood of receiving single-agent versus multiagent chemotherapy protocols at the EOL. The analysis revealed that longer disease duration was significantly associated with an increased probability of receiving less aggressive treatment, such as single-agent chemotherapy regimen. Patients with a disease duration of 3.5 years or longer had an odds ratio (OR) of 4.7 for single agent protocol compared with those with the duration of less than 1 year (95% CI, 2.33-9.28, *P* =.0005) and OR of 1.92 compared with disease duration of 1-3.4 years (95% CI, 1.0-3.7, *P* =.05). Additionally, tumor origin significantly influenced the likelihood of single-agent chemotherapy. Patients with lung cancer and GI malignancies were more likely to receive single-agent compared with those with gynecologic cancers (OR = 2.95, 95% CI, 1.08-8.06, *P* =.035; OR = 1.20, 95% CI, 0.47-3.07, *P* =.036, respectively). In contrast, age, gender, marital status, nationality, and ECOG score did not demonstrate a significant association with single-agent treatment receipt.

### Predictors of anticancer treatment administration at EOL

Using multivariate model, the probability of receiving oncologic treatment within the 30 days preceding death was significantly influenced by 4 independent clinical variables: ECOG PS score, disease duration, age, and tumor origin (Table 3). Patients with ECOG performance status score of 0-2 had an OR of 1.81 relative to those with scores of 3-4 (95% CI, 1.33-2.44, *P* <.0001). Additionally, patients with a disease duration of less than 1 year exhibited markedly higher probabilities of receiving treatment, with ORs of 2.92 (95% CI, 2.29-3.72, *P* =.0127) and 4.95 (95% CI, 3.61-6.80, *P* <.0001) when compared with individuals with durations of 1-3.4 years and 3.5 years or more, respectively. Younger patients had higher probability for treatment at EOL, patients under 55 years old and 55-74 had ORs of 1.75 (95% CI, 1.28-2.41, *P* <.0001) and 1.66 (95% CI, 1.28-2.15, *P* <.0001) in comparison to 75 years and older, respectively. Furthermore, patients receiving ICIs had 45% higher probability for treatment at 30 days before death compared with patients treated with chemotherapy (OR 1.45, 95% CI, 1.06-2.00, *P* =.02). Breast cancer patients had over twice the probability of receiving systemic anticancer treatment in the last 30 days compared with individuals with GI, GU, and lung origin (OR 2.02, 2.55, and 2.14, respectively) and a 4-fold greater risk compared with those with CNS malignancies (OR 4.01, 95% CI, 2.05-7.84, *P* <.0001). Treatment administration in the last 90 days before death was similarly influenced by shorter disease duration, younger age at diagnosis and tumor origin, with breast cancer consistently exhibiting higher treatment rates compared with GI, GU, and lung cancers (Supplementary Table 1).

**Table 3.** Odds ratios for probability of receiving treatment 30 days before death.

Independent variable	Comparison categories	Odds ratio	95% CI	*P*-value
**ECOG**	0-2 vs 3-4	1.81	(1.33, 2.44)	**.0001**
**Disease duration**	<1 year vs 1-3.4 years	2.92	(2.29, 3.72)	**.0127**
	<1 year vs ≥3.5 years	4.95	(3.61, 6.8)	**<.0001**
	1-3.4 years vs ≥3.5 years	1.698	(1.28, 2.26)	**.0003**
**Age group**	< 55 vs 55-74	1.06	(0.81, 1.38)	**.0345**
	55-74 vs ≥75	1.66	(1.28, 2.15)	**.0001**
	< 55 vs 75+	1.75	(1.28, 2.41)	**<.0001**
**Treatment type**	mAB vs mAB + ICI	4.59	(1.44, 14.49)	**.013**
	ICI vs mAB	1.09	(0.62, 1.94)	**.0037**
	mAB + CT vs mAB + ICI	3.48	(1.17, 10.39)	**.0252**
	ICI vs CT	1.45	(1.06, 2.00)	**.02**
**Diagnosis**	Breast vs GI cancer	2.02	(1.32, 3.11)	**.0022**
	GI vs CNS Cancer	1.98	(1.05, 3.73)	**.0039**
	Head & Neck vs GI cancer	1.99	(1.07, 3.7)	**.0331**
	Breast vs GU cancer	2.55	(1.44, 4.5)	**.0013**
	Head & Neck vs GU cancer	2.5	(1.23, 5.1)	**.0116**
	Breast vs CNS cancer	4.01	(2.05, 7.84)	**<.0001**
	Breast vs Lung cancer	2.14	(1.32, 3.45)	**.002**
	GYN vs CNS Cancer	2.5	(1.2, 5.2)	**.014**
	Head & Neck vs CNS cancer	3.94	(1.7, 9.1)	**.0014**
	Head & Neck vs. Lung cancer	2.1	(1.1, 4.02)	**.0253**
**Gender**	Male vs Female	-	-	.646
**Marital status**	Single vs Married	-	-	.95
**Nationality**	Jewish vs Arab	-	-	.89

Multivariate logistic regression analysis examining factors influencing the probability of receiving oncologic treatment within 30 days before death.

Abbreviations: GI, gastrointestinal; GU, genitourinary; CNS, central nervous system; CT, chemotherapy; ICIs, immune checkpoint inhibitors; mAB, monoclonal antibodies. Values that are statistically significant (*P* ≤ 0.05) are indicated in bold.

### Rate of treatment administration

**Figure 1.**
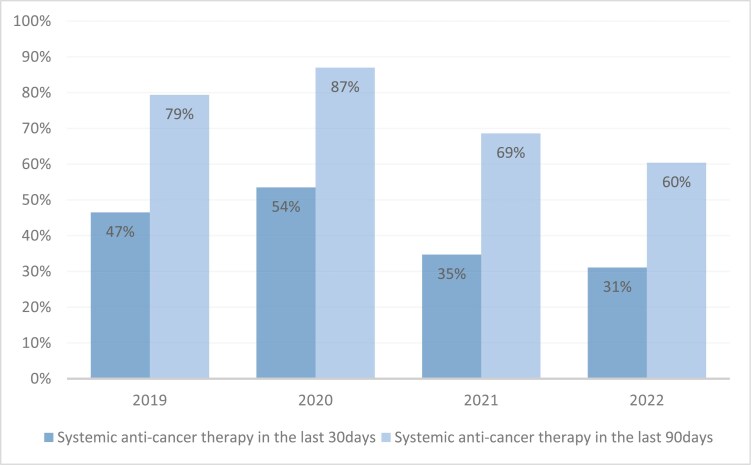
Rate of systemic anticancer therapy administration in last 30 and 90 days of life, by year.

In a year-by-year analysis (Figure 1), the lowest rates of EOL SACT in both groups (last 30 days and 90 days) was in 2022 (31.1% and 60.4%, respectively), and the highest rate were in 2020 (53.5%, 87%).

### Adjustments and initiations treatment

**Figure 2.**
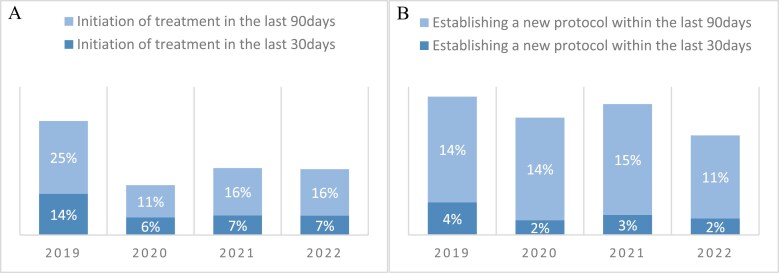
Rate of first-line therapy initiation at last 30 days and last 90 days of life, by year (A), rate of initiation of subsequent line of treatment at the last 30 and 90 days of life, by year (B).

During the last 30 days of their lives, 50 patients (2.7%) had their treatment protocol modified, while 141 (7.6%) patients began receiving systemic anticancer treatment for the first time. In the last 90 days of life, 244 (13.2%) patients underwent a change in their treatment protocol, and 312 (16.8%) initiated systemic anticancer therapy for the first time during this period. Figure 2 illustrates trends regarding the establishment of new protocols and the initiation of treatment among patients in the final 30 and 90 days of life, categorized by year.

### Performance status and aggressiveness of care

Analysis of systemic anticancer therapy administration by categories of ECOG performance status revealed that patients with performance status 0 or 1 had, as expected, the highest rates of receiving treatment (Table 4). Specifically, of all patients with performance status 0, 41% received systemic therapy in the last 30 days before their death an 68% in the last 90 days before their death. For patients with performance status 1, the percentages were 42% and 71%, respectively, for the same time periods.

**Table 4.** Distribution of patients receiving treatment according to ECOG performance status category, in relation to the total patient count in each category.

ECOG performance status	Total number of patients	Treatment rates in the last 30 days % (*N*)	Treatment rates in the last 90 days % (*N*)
0	310	41% (127)	68% (212)
1	836	42% (348)	71% (593)
2	351	32% (111)	65% (229)
3	242	26% (62)	64% (156)
4	66	27% (18)	59% (39)
NA	47	0 (0)	49% (23)

## Discussion

The findings of this study provide critical insights into the patterns of systemic anticancer treatment administration at the EOL, particularly in light of the evolving landscape of oncologic therapies. Among the 1851 eligible patients, a significant number received treatment in the final phases of their lives, highlighting a trend toward continued oncologic interventions even when the primary goal shifts to palliative care.

Notably, 36% of patients received systemic anticancer treatment within the last 30 days before death, nearly two-thirds (67.6%) of patients received treatment in the last 90 days before death, with chemotherapy being the most common modality in both time points. The study revealed that ECOG performance status, disease duration, age, and tumor origin were significant predictors of treatment likelihood. Higher rates of treatment were observed in younger aged patients, with an ECOG score of 0-2, and those with shorter disease durations, emphasizing the complex relationship between health status and therapeutic decisions at the EOL.

The observed rate of 36% of patients receiving SACT within 30 days of death in this study was notably higher than most previously reported rates, which ranged from 2.2% to 22.5% in various international studies.^4,6,16-20,22^ However, this rate aligns closely with Canavan et al.’s findings of 39% and Auclair et al.’s report of 38%, suggesting that higher rates of EOL SACT administration may be more common in recent years, particularly with the advent of immunotherapy and targeted treatments.^12,21^ The rate of systemic treatment in the last 90 days before death was 67.6%, consistent with literature ranging from 56% to 67%.^7,31^ Possible explanation to the more aggressive treatment given at our institution could be the central location with high population density allowing easy access to healthcare, availability of advanced treatments subsidized by the state, religious and cultural norms emphasizing fighting the disease, and simultaneous provision of palliative treatments alongside anticancer therapies.

In this study, 40.9% of breast cancer patients received IV anticancer treatment within 30 days of death. These patients exhibited significantly higher treatment rates, with OR ranging from 2.02 to 4.01 compared with other cancer types such as lung, CNS, GU, and GI malignancies. This finding is consistent with existing literature that indicates high rates of aggressive EOL treatment for metastatic breast cancer.^32^ However, our study excluded patients receiving oral therapies, which may have unintentionally led to the inclusion of individuals with more aggressive disease subtypes such as hormone-resistant, HER2-positive, and triple-negative breast cancer. This population often experiences shorter disease durations and is generally younger in age.

Albeit the observed inverse correlation between ECOG PS score and utilization of EOL anticancer treatment, which aligns with the ASCO guidelines against administration of anticancer treatment in patients with poor performance status.^14,15^ Over one-quarter of patients with ECOG PS score of 3 and 4 (26% and 27%, respectively) received treatment in the last 30 days of life. Yet, only 16.6% (308 patients) of our study population had ECOG PS score of 3-4. These high rates of oncologic treatment administration could be influenced by the relatively small sample size, but together with the generally high rates of treatment among the entire study population, most likely suggest an aggressive approach with a significant financial burden on the health care system^13^ and probably futile regarding quality of life.^33,34^

Following the outbreak of the COVID-19 epidemic, studies have demonstrated a correlation between cancer morbidity and an increased risk of mortality from the coronavirus.^35^ This finding helps to explain the relationship observed in our study between the administration of anticancer treatment at EOL and the year in which the treatment was administered. With 2020, the year of epidemic break, exhibiting the highest rates of therapy during both timepoints examined (last 30 and 90 days of ones’ life). This trend is likely attributed to the fact that patients who died during the epidemic year were in a more critical condition and sought treatment in hospitals despite the imposed restrictions.

Despite the global trend toward increased use of ICIs and TTs near the EOL, often as part of precision medicine guided by tumor molecular profiling and following standard-of-care treatments, along with a decline in chemotherapy use,^5,12^ in our study, chemotherapy remained the most commonly administered treatment at EOL. A possible explanation could be the subsidy of treatment. In cases when novel “expansive” oncologic medications are not provided by the national health care basket, chemotherapy is typically subsidized and offered free of charge. With regards to cultural norms such as fighting the disease, personal views and values, and general difficulty of therapy cessation, choices of patients and caregivers around the EOL are not a consequence of medical decisions based on evidence-based medicine alone. Therefore, most patients continue chemotherapy provided earlier and 10% even started a new line of treatment at 30 days from death.

It is vital to recognize that the evidence does not conclusively support universal early treatment withdrawal, as some patients may still benefit from less aggressive oncologic therapies. The key challenge lies in identifying the potentially small subset of patients who could derive such benefit. In our study, multiagent chemotherapy protocols dominated (69.6%) in patients’ final 30 days of life. Notably, patients with longer disease trajectories and specific tumor types, particularly lung and GI malignancies, showed a higher likelihood of receiving less aggressive single-agent therapy. These findings emphasize the critical importance of nuanced patient selection and personalized treatment strategies during EOL care.

Palliative care in Israel has progressed significantly since its first modern hospice in 1983, with legislative support in 2005 and recognition as a subspecialty in 2012. Despite these advancements, a considerable gap remains between available services and patient needs. In 2015, a palliative care unit was established at Tel-Aviv Sourasky Medical Center, gaining recognition as a specialization center in 2022. The unit is closely integrated with the Oncology division. However, our study’s lack of hospice care implementation data limits our understanding of its impact on palliative and oncologic outcomes.

The study’s limitations, include those arising from its design being retrospective, the study was limited to a single medical center and the high level of ethnic homogeneity of the study population. Additionally, it is important to consider the timing of data collection, which coincided with the outbreak of the COVID-19 epidemic. During this period, patients may have reduced their in-person interactions, resulting in decreased meetings with the healthcare team and potential alterations in treatment receipt. This could introduce confounding factors that might influence the outcomes observed. Our study has several strengths—the large sample size, the collection of extensive demographic data, and the participation of patients who were treated by multiple doctors, who provided different insight and took treatment decisions, an advantage arising from the fact that TLVMC is a tertiary medical center.

In conclusion, our study demonstrates that, in the absence of regulatory measures or financial constraints, aggressive EOL treatments are likely to persist. Awareness of different treatment options along the way is imperative for healthcare providers, and acknowledgment of the concept of “a good death” is essential when making decisions for terminal cancer patients. Additionally, we suggest that health care professionals around the world continue to learn, research, and ask questions about the necessity of anticancer therapy at EOL, to the individual patient and to the system as a whole. Our results may imply that EOL SACT do not meet doctors’ expectations as well as patients’ care-wishes.

## Supplementary Material

oyaf066_suppl_Supplementary_Tables_1

## Data Availability

The data underlying this article will be shared on reasonable request to the corresponding author. A link to data depository: https://drive.google.com/drive/folders/14lFwuSZdlLs7ddtQtPvo_qmHU9F2vPd3?usp=sharing
