# Supplementary material for: Systemic anticancer therapy at the end of life: real-world insights from a tertiary oncology center in Israel
Source: Oncologist. 2025 May 16;30(5):oyaf066. doi: 10.1093/oncolo/oyaf066 (PMC12082831; doi:10.1093/oncolo/oyaf066)
Supplement: oyaf066_suppl_Supplementary_Tables_1 [file oyaf066_suppl_supplementary_tables_1.docx]

Supplementary Table 1: Odds Ratios for Probability of Receiving Treatment 90 Days Before Death

| **Independent Variable** | **Comparison Categories** | **Odds Ratio** | **95% Confidence Interval** | **P-value** |
| --- | --- | --- | --- | --- |
| **Disease Duration** | - | - | - | **<0.0001** |
|  | <1 year vs. 1-3.4 years | 4.69 | (3.48, 6.33) | **0.0002** |
|  | <1 year vs. ≥3.5 years | 9.71 | (6.9, 13.7) | **<0.0001** |
|  | 1-3.4 years vs. ≥3.5 years | 2.07 | (1.61, 2.66) | **<0.0001** |
| **Age Group** | - | - | - | **0.0119** |
|  | 55-74 vs. ≥75 | 1.36 | (1.05, 1.76) | **0.0214** |
|  | <55 vs. ≥75 | 1.64 | (1.19, 2.27) | **0.0025** |
|  | 55-74 vs. <55 | - | - | 0.59 |
| **Diagnosis** | - | - | - | **0.0001** |
|  | Breast vs. GI cancer | 1.76 | (1.14, 2.7) | **0.0214** |
|  | GI vs. GU cancer | 1.36 | (0.96, 2.05) | **0.0124** |
|  | GI vs. Lung cancer | 1.33 | (0.97, 1.83) | **0.0024** |
|  | Breast vs. GU cancer | 2.39 | (1.4, 4.1) | **0.0015** |
|  | GYN vs. GU cancer | 2.07 | (1.19, 3.61) | **0.0098** |
|  | Head & Neck vs. GU cancer | 2.61 | (1.25, 5.46) | **0.011** |
|  | Breast vs. Lung cancer | 2.34 | (1.4, 3.8) | **0.0006** |
|  | GYN vs. Lung cancer | 2.03 | (1.24, 3.33) | **0.0052** |
|  | Head & Neck vs. Lung cancer | 2.55 | (1.27, 5.11) | **0.0083** |
| **Gender** | - | - | - | 0.69 |
| **Marital Status** | - | - | - | 0.19 |
| **Nationality** | - | - | - | 0.38 |
| **Treatment Type** | - | - | - | 0.21 |
| **ECOG** | - | - | - | 0.06 |

Multivariate logistic regression analysis examining factors influencing the probability of receiving oncologic treatment within 90 days before death.

Abbreviations: GYN, gynecologic.
